# Identification of novel differentially expressed genes in retinas of STZ‐induced long‐term diabetic rats through RNA sequencing

**DOI:** 10.1002/mgg3.1115

**Published:** 2020-01-20

**Authors:** Xindan Xing, Yan Jiang, Hanying Wang, Yuan Zhang, Tian Niu, Yuan Qu, Chingyi Wang, Haiyan Wang, Kun Liu

**Affiliations:** ^1^ Department of Ophthalmology Shanghai General Hospital National Clinical Research Center for Eye Diseases Shanghai Key Laboratory of Ocular Fundus Diseases Shanghai Engineering Center for Visual Science and Photomedicine Shanghai Engineering Center for Precise Diagnosis and Treatment of Eye Diseases Shanghai Jiao Tong University School of Medicine Shanghai China

**Keywords:** diabetic retinopathy, differentially expressed genes, GO enrichment analysis, KEGG pathway analysis, RNA sequencing

## Abstract

**Background:**

The aim of this research was to investigate the retinal transcriptome changes in long‐term streptozotocin (STZ)‐induced rats' retinas using RNA sequencing (RNA‐seq), to explore the molecular mechanisms of diabetic retinopathy (DR), and to identify novel targets for the treatment of DR by comparing the gene expression profile we obtained.

**Methods:**

In this study, 6 healthy male SD rats were randomly divided into wild‐type (WT) group and streptozotocin (STZ)‐induced group, 3 rats each group. After 6 months, 3 normal retina samples and 3 DM retina samples (2 retinas from the same rat were considered as 1 sample) were tested and differentially expressed genes (DEGs) were measured by RNA‐seq technology. Then, we did Gene Ontology (GO) enrichment analysis and KEGG (Kyoto Encyclopedia of Genes and Genomes) pathway analysis and validated the results of RNA‐seq through qRT–PCR.

**Results:**

A total of 118 DEGs were identified, of which 72 were up‐regulated and 46 were down‐regulated. The enriched GO terms showed that 3 most significant enrichment terms were binding (molecular function), cell part (cellular component), and biological regulation (biological process). The results of the KEGG pathway analysis revealed a significant enrichment in cell adhesion molecules, PI3K‐Akt signaling pathway, and allograft rejection, etc.

**Conclusion:**

Our research has identified specific DEGs and also speculated their potential functions, which will provide novel targets to explore the molecular mechanisms of DR.

## Graphical abstract

This study examined retina from WT and diabetic SD male rats to investigate the changes in a variety of retinal transcripts as a result of diabetes using RNA‐seq. We identified a total of 118 DEGs, of which 72 were up‐regulated and 46 were down‐regulated. We also found 66 GO terms and 41 KEGG pathways which were significantly enriched by GO and KEGG analysis.

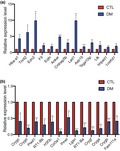

## 1 INTRODUCTION

With the notably increasing population of diabetes, diabetic retinopathy (DR), one of the most severe complications of diabetes, has the potential to be the leading cause of blindness worldwide (Klein, 2007; Yau et al., 2012). The main pathological change of DR is the retinal capillary endothelial damage. Existing researches have revealed that the major pathogenesis of DR includes susceptibility genes, increased polyol pathway flux, increased advanced glycation end‐product (AGE) formation, abnormal activation of protein kinase C (PKC) pathway, and increased hexosamine pathway flux (Frank, 2004). These pathways can cause up‐regulation of factors such as insulin‐like growth factor (*IGF,* OMIM: 147440), vascular endothelial growth factor (*VEGF*, OMIM: 192240), tumor necrosis factor (*TNF*, OMIM: 191160), and basic fibroblast growth factor‐2 (*bFGF‐2* OMIM: 134920) that can promote the occurrence of DR (Safi, Qvist, & Kumar, 2014). However, the mechanisms of DR are really complex, and the internal relations between pathways and biomarkers are complicated (Saxena, Singh, & Saklani, 2016). Identifying new significantly differentially expressed genes (DEGs) and speculating their roles might help us to better understand the molecular network of the pathogenesis of DR and provide new research ideas.

Streptozotocin (STZ) is a pancreatic islet β‐cell‐cytotoxic antibiotic, which can highly and selectively destroy pancreatic islet B cells. So it has been widely used to develop animal models of human condition with either type 1 diabetes mellitus or type 2 diabetes rat mellitus (Furman, 2015).

Previous RNA‐seq studies of DR mostly use 2 months course diabetic rats, but streptozotocin‐induced diabetic retinopathy in rats might not be apparent within 2 months as the previous paper showed (Chen, Bin, & Qian, 2013; Yuan, Zhi, & Geng, 2016). In this study, we analyzed whole transcriptome of mRNA in retina from both normal and 6 months diabetes mellitus (DM) male Sprague Dawley (SD) rats, identified differentially expressed genes, and also speculated their roles based on Illumina HiSeq sequencing platform combining with Gene Ontology (GO) and KEGG (Kyoto Encyclopedia of Genes and Genomes) pathway enrichment analysis. We hope our study will enhance our understanding of the molecular mechanisms underlying the pathogenesis of DR and allow the development of novel therapeutic targets of DR.

## 2 METHODS

### 2.1 Ethical compliance

The experimental protocols used in this study followed guidelines established by the ARVO Statement for the Use of Animals in Ophthalmic and Vision Research and were approved by the Ethics Committee of Shanghai General Hospital, Shanghai Jiaotong University, Shanghai, China (Permit Number: 2009‐0086).

### 2.2 Animals

Adult male SD rats (250–300 g) were obtained from the Shanghai Laboratory Animal Center. Healthy male SD rats were randomly divided into wild‐type (WT) group and STZ‐induced group. STZ (Sigma‐Aldrich) was injected in rats' abdomen. Rats in WT group received an intraperitoneal injection of citrate buffer only. Diabetes was confirmed by assaying the glucose concentration in blood collected from the tail vein using a precision glucometer (Roche Diabetes Care GmbH) weekly after STZ injection. Rats with blood glucose concentration >300 mg/dl were considered diabetic. Rats were sacrificed by intraperitoneal injection of excess 10% chloral hydrate (10 ml/kg). All rats had received cardiac perfusion using physiological saline to remove blood from retinas before they sacrificed 6 months after the injection of STZ.

### 2.3 mRNA sequencing by illumina Hiseq

#### 2.3.1 RNA‐seq

Total RNA of each sample was extracted using Trizol. Total RNA of each sample was quantified and qualified by Agilent 2100 Bioanalyzer (Agilent Technologies), NanoDrop (Thermo Fisher Scientific Inc.), and 1% agarose gel. 1 μg total RNA with RIN value above 7 was used for following library preparation. Next‐generation sequencing library preparations were constructed according to the manufacturer's protocol (NEBNext^®^ UltraTM RNA Library Prep Kit for Illumina^®^). The poly(A) mRNA isolation was performed using NEBNext Poly(A) mRNA Magnetic Isolation Module (NEB) or Ribo‐ZeroTM rRNA removal Kit (Illumina). The mRNA fragmentation and priming were performed using NEBNext First Strand Synthesis Reaction Buffer and NEBNext Random Primers. First‐strand cDNA was synthesized using ProtoScript II reverse transcriptase, and the second‐strand cDNA was synthesized using Second Strand Synthesis Enzyme Mix. The purified double‐stranded cDNA (by AxyPrep Mag PCR Clean‐up (Axygen) was then treated with End Prep Enzyme Mix to repair both ends and add a dA‐tailing in one reaction, followed by a T‐A ligation to add adaptors to both ends. Size selection of Adaptor‐ligated DNA was then performed using AxyPrep Mag PCR Clean‐up (Axygen), and fragments of ~360 bp (with the approximate insert size of 300 bp) were recovered. Each sample was then amplified by PCR for 11 cycles using P5 and P7 primers, with both primers carrying sequences which can anneal with flow cell to perform bridge PCR and P7 primer carrying a six‐base index allowing for multiplexing. The PCR products were cleaned up using AxyPrep Mag PCR Clean‐up (Axygen), validated using an Agilent 2100 Bioanalyzer (Agilent Technologies), and quantified by Qubit 2.0 Fluorometer (Invitrogen). Then, libraries with different indices were multiplexed and loaded on an Illumina HiSeq instrument according to manufacturer's instructions (Illumina). Sequencing was carried out using a 2 × 150 bp paired‐end (PE) configuration; image analysis and base calling were conducted by the HiSeq Control Software (HCS) + OLB + GAPipeline‐1.6 (Illumina) on the HiSeq instrument. The sequences were processed and analyzed by GENEWIZ.

#### 2.3.2 Data analysis

##### Quality control

In order to remove technical sequences, including adapters, polymerase chain reaction (PCR) primers or fragments thereof, and quality of bases lower than 20, pass filter data of fastq format were processed by Trimmomatic (v0.30) into high‐quality clean data.

##### Mapping

Firstly, reference genome sequences and gene model annotation files of relative species were downloaded from genome website, including UCSC, NCBI, ENSEMBL. Secondly, Hisat2 (v2.0.1) was used to index reference genome sequence. Finally, clean data were aligned to reference genome via software Hisat2 (v2.0.1).

##### Expression analysis

In the beginning, transcripts in fasta format were converted from known gff annotation file and indexed properly. Then, with the file as a reference gene file, HTSeq (v0.6.1) estimated the expression level of genes and isoforms from the pair‐end clean data.

##### Differential expression analysis

The DESeq Bioconductor package, a model based on the negative binomial distribution, was used in the differential expression analysis. Benjamini and Hochberg's approach was applied to control the false discovery rate, and genes with *p*‐value <.05 were considered differently expressed.

##### GO and KEGG enrichment analysis

GO‐TermFinder was used to identify GO terms that annotate a list of enriched genes with a significant *p*‐value less than.05.

KEGG is a collection of databases dealing with genomes, biological pathways, diseases, drugs, and chemical substances (http://en.wikipedia.org/wiki/KEGG). We used scripts in‐house to enrich significant differential expression gene in KEGG pathways.

### 2.4 Quantitative real‐time PCR

Total RNA was isolated with Trizol and reverse‐transcribed into cDNA using the PrimeScript RT Master Mix kit (Takara Bio). The expression of specific genes was quantitated by real‐time PCR using SYBR Premix Ex Taq kit (Takara Bio) on an ABI 7500HT machine (Applied Biosystems) with 18sRNA (rat) as internal control. Primers used in qRT‐PCR are listed in Table 1.

**Table 1:** List of primers used in qRT–PCR

Gene	Primer sequence (5′−3′)
Asb15	F:TGAAATACGGAGGAAGTG
	R:ATCTAAGTTGGGGTCTGC
Ltk	F:GGCTGATGGGGAAGATGG
	R:AGAGGGCTTGTGGTGGTT
Lad1	F:ACCACGAGGGAGGTCAG
	R:ACGGGTAGGAAGCGAGA
Tnnt2	F:GGAAGACTGGAGCGAAGA
	R:AAGTTGGGCATGAAGAGC
Eqtn	F:GACAGCCACAACTGACCT
	R:GTGAGATTCCCAGCATTA
Hba‐a1	F:GCTCACGGCAAGAAGGTT
	R:TGCTCACAGAGGCAAGGA
Ankar	F:ACCTGATTCTTCCCTACA
	R:CAGTTTCATCCACCACA
F9	F:TCCTTCTGGGCTATCTAC
	R:AAAACTTCTCGTGCTTCT
Impad1	F:CGACGAGGTGAGACGAGT
	R:CATCAAGTGGGTCAATCC
Tsga10ip	F:GCAGGGCAGACAACAAA
	R:TCAGCCTCGTCAGCATT
Edn2	F:GACCCCACCTGCGTTTTC
	R:ATTTCCTCCCGTCTCCAC
Cntnap5b	F:CTTTGGTGCTGTCTGGT
	R:TCTCATGCCTATCTTTCC
Col3a1	F:CACCTGCTCCTGTCATTCC
	R:CCATTCCTCCGACTCCAGA
Fam111a	F:CAAGCATCTACCAATTATG
	R:TCATCAACTGGCTGAGTGT
H3f3c	F:AGACTGCCCGCAAATCCAC
	R:CGCACCAGACGCTGAAAG
Pmel	F:GACTGTGGACTGCTCCGTG
	R:CCTCCGCTCTTAGGATTGA
Piwil1	F:TCAGACTCGGAATGGGGAA
	R:ATCAATCGGGTCACTTGGG
Cryga	F:CTGTCAACAACGATGGGAA
	R:TCCACTGCTGGTAGTCAGG
Lgsn	F:CTTTTATTACCTGCTGGAT
	R:ATCGTTCATGTCAACCGTT
RT1‐Ba	F:GAAACAGCAAGCCAGTCA
	R:GAATCTCAGGTTCCCAGTG
RT1‐Bb	F:GCTTATTAGGAACGGGGAC
	R:TTCTGACGCTTGTGACGGA
Crygf	F:CAGATGGTGGAGATCACAG
	R:TGATGAAAGGGCAGAGTAA
Crygd	F:ACCAGCAGTGGATGGGTTT
	R:TCCAGCACATTGAGGGAG
Crygb	F:AGTGGCTGCTGGATGCTCT
	R:TCTGTAAGTGCCCGAGTGT
Crygc	F:CAAAGGCGTCATGATGGAG
	R:TATAAATCTACCACCCTCC
18sRNA	F:GTTGAACCCCATTCGTGAT
	R:AGCTTATGACCCGCACTTA

Abbreviations: F, forward; R, reverse.

### 2.5 GenBank reference sequence and version number

Hba‐a1: Gene ID: 287167, Refseq: NC_005109.4

Tnnt2: Gene ID: 24837, Refseq: NC_005112.4

Edn2: Gene ID: 24324, Refseq: NC_005104.4

F9: Gene ID: 24946, Refseq: NC_005120.4

Eqtn: Gene ID: 500502, Refseq: NC_005104.4

Ankar: Gene ID: 501138, Refseq: NC_005108.4

Cntnap5b: Gene ID:301650, Refseq: NC_005112.4

Lad1: Gene ID: 313325, Refseq: NC_005112.4

Asb15: Gene ID: 500050, Refseq: NC_005103.4

Tsga10ip: Gene ID:361707, Refseq: NC_005100.4

Ltk: Gene ID:311337, Refseq: NC_005102.4

Impad1: Gene ID: 312952, Refseq: NC_005116.4

Loxhd1: Gene ID: 291427, Refseq: NC_005117.4

Crygc: Gene ID: 24277, Refseq: NC_005108.4

Crygd: Gene ID: 24278, Refseq: NC_005108.4

Piwil1: Gene ID: 363912, Refseq: NC_005111.4

RT1‐Bb: Gene ID: 309622, Refseq: NC_005119.4

H3f3c: Gene ID: 100360868, Refseq: NC_005106.4

Col3a1: Gene ID: 84032, Refseq: NC_005108.4

Pmel: Gene ID: 362818, Refseq: NC_005106.4

Lgsn: Gene ID: 316304, Refseq: NC_005108.4

RT1‐Ba: Gene ID: 309621, Refseq: NC_005119.4

Crygf, Gene ID: 689947, Refseq: NC_005108.4

Cryga: Gene ID: 684028, Refseq: NC_005108.4

Fam111a, Gene ID: 499322, Refseq: NC_005100.4

Fgf2: Gene ID: 54250, Refseq: NC_005101.4

Dmrtb1: Gene ID: 313484, Refseq: NC_005104.4

## 3 RESULTS

### 3.1 General RNA‐seq analysis

**Figure 1:**
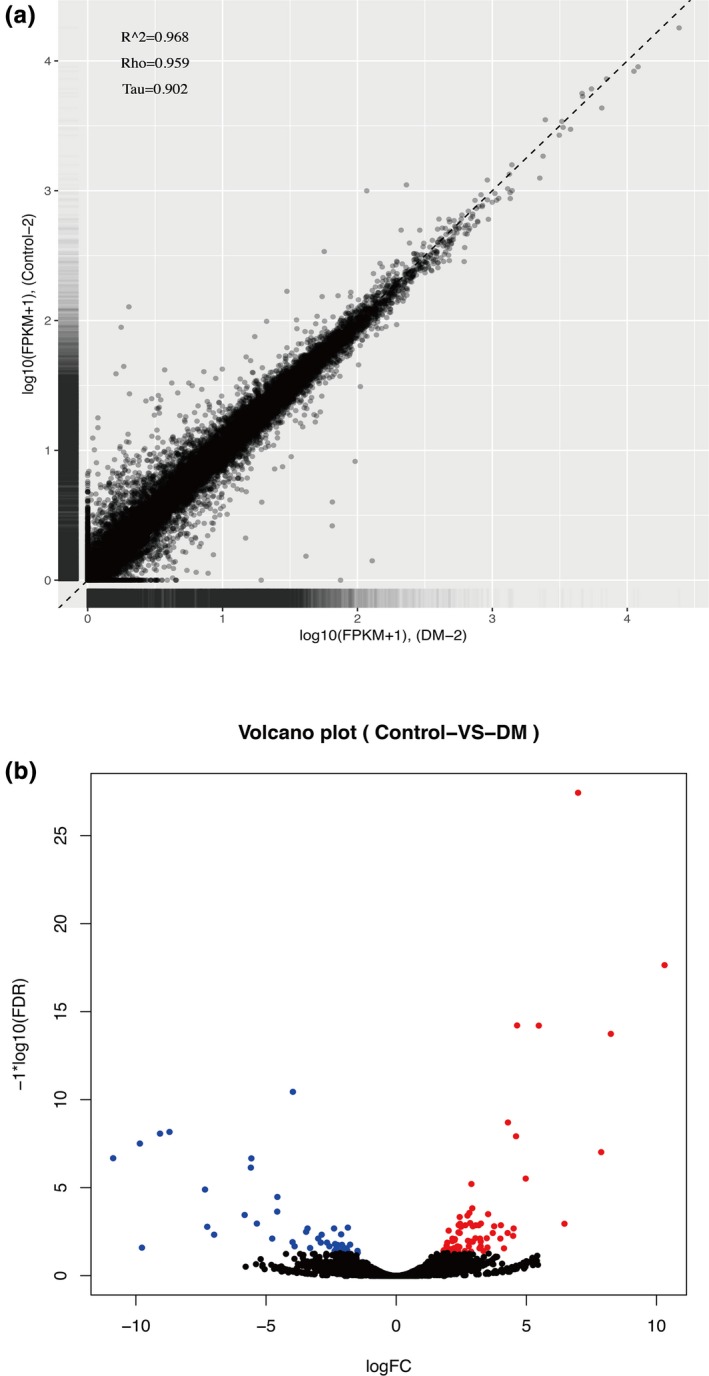
General RNA‐seq analysis. (a) The correlation test between samples. (b) Volcano plot of DEGs. Blue dots represent the number of down‐regulated genes, red dots represent the number of up‐regulated genes, and black dots represent the genes which are not DEGs

Six pairs of retina samples from 3 normal rats and 3 diabetic SD male rats were sequenced 3 times. In total, we established six RNA‐seq libraries and obtained over 45,000,000 clean reads in each library, in which more than 90% clean reads can be mapped to the reference genome. The detailed mapping data are listed in Table 2. The correlation test between samples verified the reliability of our test and the rationality of sample selection: control 1/DM1 group: *R* ^2^ = .834, control 2/DM2 group, *R* ^2^ = .968 (Figure 1a), control 3/DM3 group: *R* ^2^ = .896, respectively. For DEGs, we identified 118 different expression genes, with 72 of them up‐regulated and 46 down‐regulated (Figure 1b). Top 10 up‐regulated and down‐regulated genes are listed in Tables 3 and 4, respectively.

**Table 2:** The filtered data quality statistics

Samples	Total reads	Total mapped	Multiple mapped	Uniquely mapped
Control‐1	45,948,036	42,033,078 (91.48%)	4,177,999 (9.09%)	37,855,079 (82.37%)
Control‐2	58,660,660	54,116,928 (92.25%)	4,353,433 (7.42%)	49,763,495 (84.83%)
Control‐3	56,556,154	52,322,394 (92.51%)	4,592,546 (8.12%)	47,729,848 (84.39%)
DM‐1	52,459,688	48,199,706 (91.88%)	3,847,104 (7.33%)	44,352,602 (84.55%)
DM‐2	53,296,824	49,070,183 (92.07%)	3,934,165 (7.19%)	45,236,018 (84.88%)
DM‐3	61,256,752	56,241,909 (91.81%)	4,460,378 (7.28%)	51,781,531 (84.53%)

Total reads: the number of clean reads; Total mapped: the number of clean reads which can locate in the genome; Multiple mapped: the number of clean reads which have more than one location on the reference sequence; Uniquely mapped: the number of clean reads which have only one location on the reference sequence.

**Table 3:** Top 10 up‐regulated genes in the retina of STZ‐induced rats, compared with normal rats

Gene ID	Gene symbol	LogFC	*p*‐value	FDR
ENSRNOG00000006365	Asb15	10.31	<.001	<0.001
ENSRNOG00000009144	Lad1	4.64	<.001	<0.001
ENSRNOG00000033734	Tnnt2	4.60	<.001	<0.001
ENSRNOG00000026323	Eqtn	4.51	<.001	0.002
ENSRNOG00000023213	Dmrtb1	4.49	<.001	0.005
ENSRNOG00000017392	Fgf2	4.29	<.001	<0.001
ENSRNOG00000045989	Hba‐a1	4.28	<.001	0.004
ENSRNOG00000003991	Ankar	4.14	<.001	0.028
ENSRNOG00000003430	F9	4.02	<.001	0.001
ENSRNOG00000027079	Impad1	4.00	<.001	0.008

GeneBank ID and version number : Asb15: Gene ID: 500050, Refseq: NC_005103.4, Lad1: Gene ID: 313325, Refseq: NC_005112.4, Tnnt2: Gene ID: 24837, Refseq: NC_005112.4, Eqtn: Gene ID: 500502, Refseq: NC_005104.4, Dmrtb1: Gene ID: 313484, Refseq: NC_005104.4, Fgf2: Gene ID: 54250, Refseq: NC_005101.4, Hba‐a1: Gene ID: 287167, Refseq: NC_005109.4, Ankar: Gene ID: 501138, Refseq: NC_005108.4, F9: Gene ID: 24946, Refseq: NC_005120.4, Impad1: Gene ID: 312952, Refseq: NC_005116.4.

**Table 4:** Top 10 down‐regulated genes in the retina of STZ‐induced rats, compared with normal rats

Gene ID	Gene symbol	LogFC	*p*‐value	FDR
ENSRNOG00000032869	Crygf	−8.71	<.001	<0.001
ENSRNOG00000032708	RT1‐Bb	−7.34	<.001	<0.001
ENSRNOG00000000451	RT1‐Ba	−6.99	<.001	0.005
ENSRNOG00000012205	Lgsn	−5.83	<.001	<0.001
ENSRNOG00000014790	Cryga	−5.59	<.001	<0.001
ENSRNOG00000000934	Piwil1	−4.57	<.001	<0.001
ENSRNOG00000023085	Pmel	−4.57	<.001	<0.001
ENSRNOG00000032401	H3f3c	−3.97	<.001	0.000
ENSRNOG00000012067	Fam111a	−3.91	<.001	0.021
ENSRNOG00000003357	Col3a1	−3.46	<.001	0.003

GeneBank ID and version number : Crygf: Gene ID: 689947, Refseq: NC_005108.4, RT1‐Bb: Gene ID: 309622, Refseq: NC_005119.4, RT1‐Ba: Gene ID: 309621, Refseq: NC_005119.4, Lgsn: Gene ID: 316304, Refseq: NC_005108.4, Cryga: Gene ID: 684028, Refseq: NC_005108.4, Piwil1: Gene ID: 363912, Refseq: NC_005111.4, Pmel: Gene ID: 362818, Refseq: NC_005106.4, H3f3c: Gene ID: 100360868, Refseq: NC_005106.4, Fam111a, Gene ID: 499322, Refseq: NC_005100, Col3a1: Gene ID: 84032, Refseq: NC_005108.4

### 3.2 GO enrichment analysis

**Figure 2:**
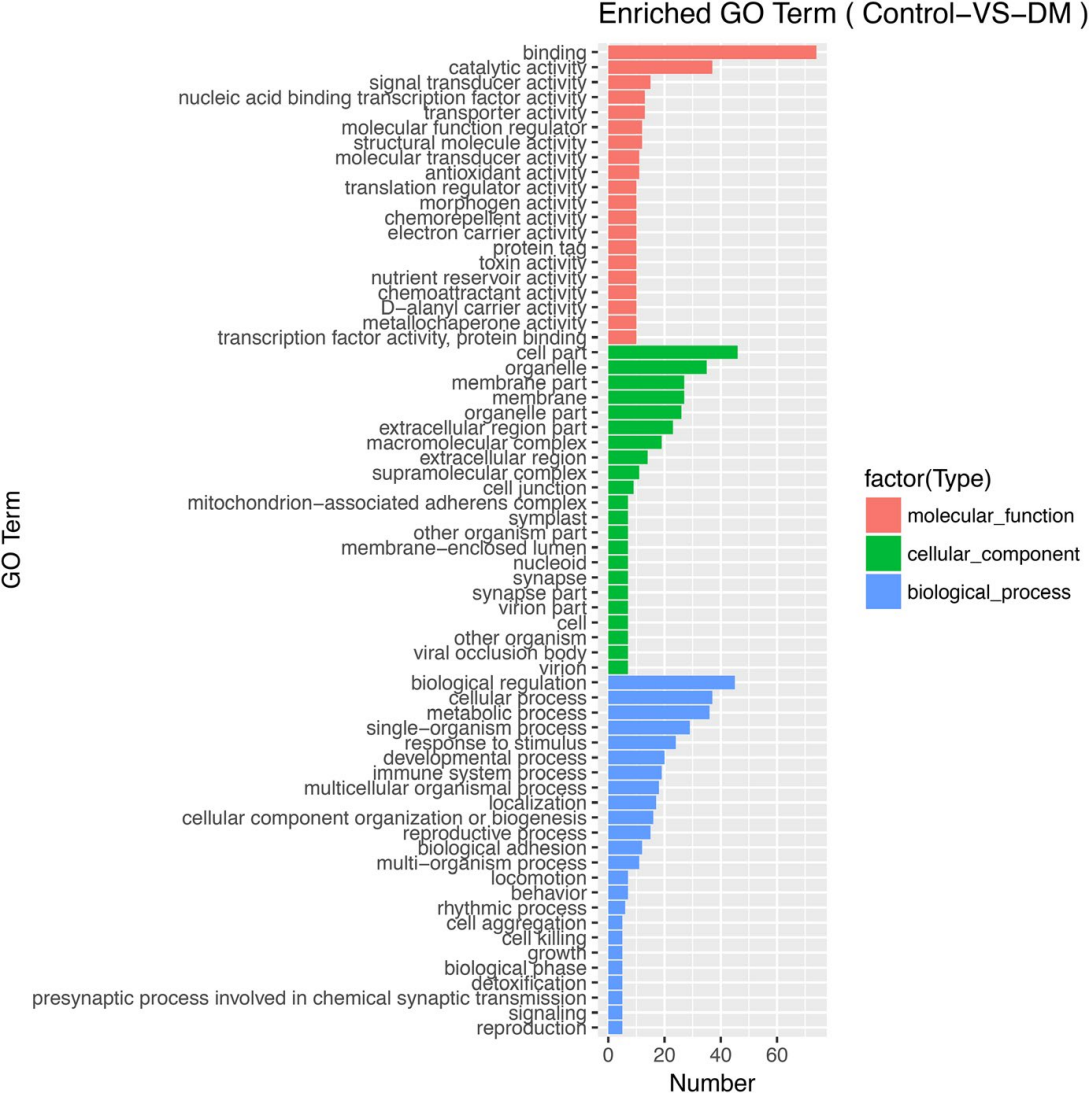
Go enrichment analysis. Red, green, and blue bars represent the enrichment of DEGs in molecular function, cellular component, and biological process, respectively

GO contains three ontologies, which are molecular function, cellular component, and biological process. In our study, we identified 66 enriched GO terms, among which 20 belong to molecular function, 22 belong to cellular component, and 24 belong to biological process. The three most enriched molecular function terms were binding, catalytic activity, and signal transducer activity. In the ontologies of cellular component and biological process, cell part, organelle, membrane part and biological regulation, cellular process, metabolic process ranked the three most enriched go terms, respectively (Figure 2).

### 3.3 KEGG pathway enrichment analysis

**Figure 3:**
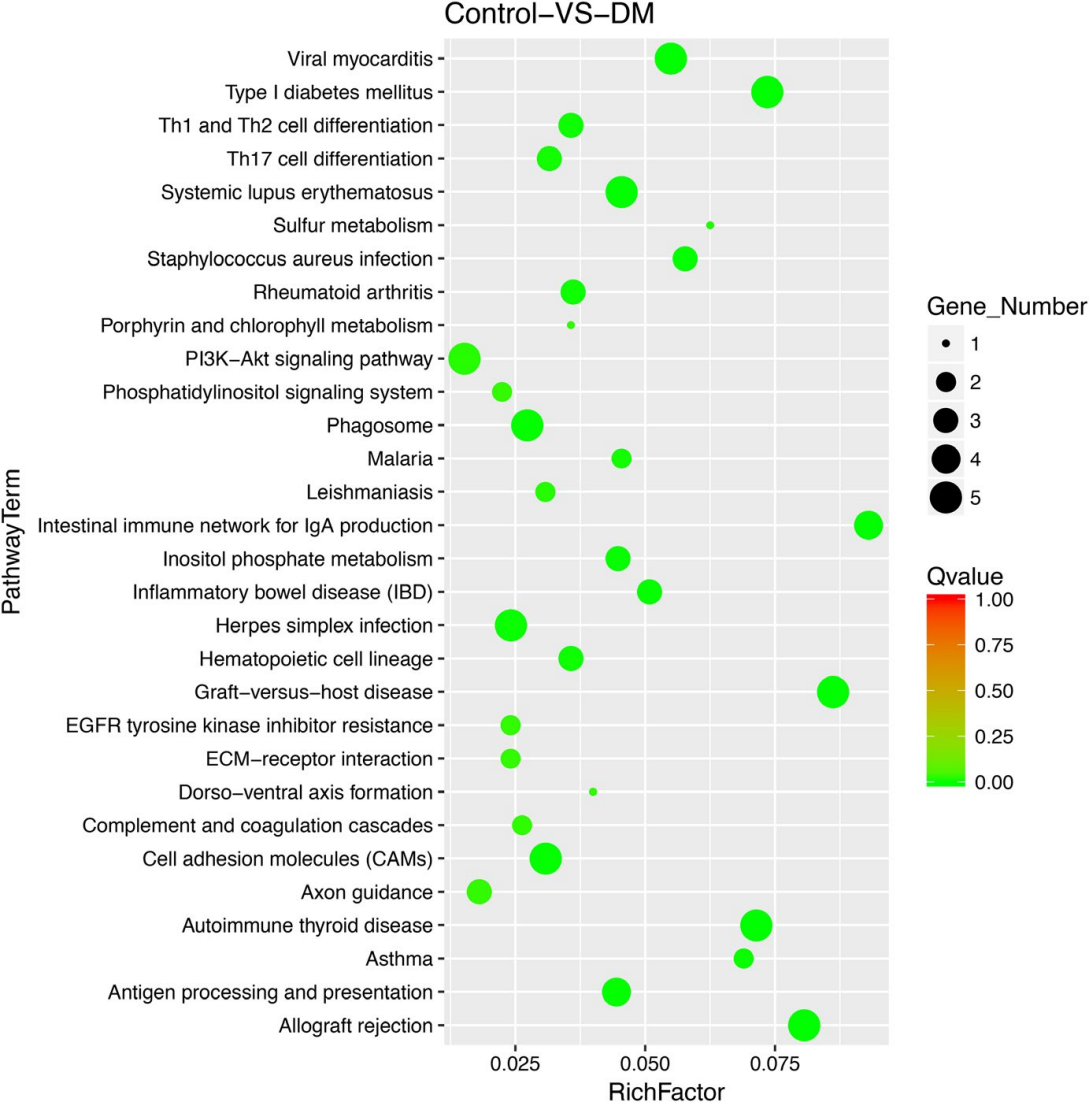
KEGG pathway analysis. The size of the circle corresponds gene number. The color corresponds *Q* value

In total, we identified 41 KEGG pathways and classified these pathways into five categories. Specifically, Human Disease category contained 20 pathways. Organismal System category contained 9 pathways. Metabolism category and Environmental Information Processing category contained 7 and 4 pathways, respectively, and Cell Processes category contained 1 pathway which named Phagosome. Thirty most enrichment items chosen for this study are shown in Figure 3. The Details are listed in Table 5, 6, 7, 8.

**Table 5:** Human Disease KEGG pathway

Pathway ID	Pathway	*Q* value	Gene list	KO list
ko04930	Type II diabetes mellitus	.049	ENSRNOG00000002946	K04696,
ko05145	Toxoplasmosis	.038	ENSRNOG00000032708, ENSRNOG00000000451	K06752,
ko05020	Prion diseases	.034	ENSRNOG00000002115	K04565
ko05146	Amebiasis	.032	ENSRNOG00000048358, ENSRNOG00000003357,	K13963, K06236
ko05164	Influenza A	.027	ENSRNOG00000032708, ENSRNOG00000000451, ENSRNOG00000002946,	K06752, K04696
ko05143	African trypanosomiasis	.027	ENSRNOG00000045989,	K13822
ko05166	HTLV‐I infection	.025	ENSRNOG00000032708, ENSRNOG00000000451, ENSRNOG00000000777, ENSRNOG00000060412,	K06752, K06751,
ko05140	Leishmaniasis	.015	ENSRNOG00000032708, ENSRNOG00000000451,	K06752,
ko05144	Malaria	.005	ENSRNOG00000012471, ENSRNOG00000045989	K04659, K13822,
ko05323	Rheumatoid arthritis	.003	ENSRNOG00000032708, ENSRNOG00000000451, ENSRNOG00000001527	K06752, K05412
ko05168	Herpes simplex infection	.002	ENSRNOG00000032708, ENSRNOG00000000451, ENSRNOG00000002946, ENSRNOG00000000777, ENSRNOG00000060412	K06752, K04696, K06751
ko05310	Asthma	.002	ENSRNOG00000032708, ENSRNOG00000000451	K06752,
ko05321	Inflammatory bowel disease (IBD)	.001	ENSRNOG00000032708, ENSRNOG00000015441, ENSRNOG00000000451	K06752, K05071
ko05150	Staphylococcus aureus infection	.001	ENSRNOG00000032708, ENSRNOG00000000451, ENSRNOG00000011971	K06752, K01331
ko05322	Systemic lupus erythematosus	<.001	ENSRNOG00000032401, ENSRNOG00000032708, ENSRNOG00000000451, ENSRNOG00000001527, ENSRNOG00000011971	K11253, K06752, K05412, K01331
ko05416	Viral myocarditis	<.001	ENSRNOG00000032708, ENSRNOG00000000451, ENSRNOG00000000777, ENSRNOG00000060412, ENSRNOG00000001527	K06752, K06751, K05412
ko05320	Autoimmune thyroid disease	<.001	ENSRNOG00000032708, ENSRNOG00000000451, ENSRNOG00000000777, ENSRNOG00000060412, ENSRNOG00000001527	K06752, K06751, K05412
ko04940	Type I diabetes mellitus	<.001	ENSRNOG00000032708, ENSRNOG00000000451, ENSRNOG00000000777, ENSRNOG00000060412, ENSRNOG00000001527	K06752, K06751, K05412
ko05330	Allograft rejection	<.001	ENSRNOG00000032708, ENSRNOG00000000451, ENSRNOG00000000777, ENSRNOG00000060412, ENSRNOG00000001527	K06752, K06751, K05412
ko05332	Graft‐versus‐host disease	<.001	ENSRNOG00000032708, ENSRNOG00000000451, ENSRNOG00000000777, ENSRNOG00000060412, ENSRNOG00000001527	K06752, K06751, K05412

**Table 6:** Organismal systems KEGG pathway

Pathway ID	Pathway	*Q* value	Gene list	KO list
ko04650	Natural killer cell‐mediated cytotoxicity	.048	ENSRNOG00000000777, ENSRNOG00000060412	K06751
ko04670	Leukocyte transendothelial migration	.038	ENSRNOG00000006860, ENSRNOG00000003866	K07363, K04189
ko04360	Axon guidance	.025	ENSRNOG00000029184, ENSRNOG00000017525, ENSRNOG00000003866	K05104, K05102, K04189
ko04320	Dorso‐ventral axis formation	.023	ENSRNOG00000000934	K02156
ko04610	Complement and coagulation cascades	.021	ENSRNOG00000003430, ENSRNOG00000011971	K01321, K01331
ko04640	Hematopoietic cell lineage	.003	ENSRNOG00000032708, ENSRNOG00000015441, ENSRNOG00000000451	K06752, K05071
ko04612	Antigen processing and presentation	.001	ENSRNOG00000032708, ENSRNOG00000000451, ENSRNOG00000000777, ENSRNOG00000060412	K06752, K06751
ko04672	Intestinal immune network for IgA production	<.001	ENSRNOG00000032708, ENSRNOG00000000451, ENSRNOG00000003866, ENSRNOG00000001527	K06752, K04189, K05412
ko04215	Apoptosis—multiple species	.037	ENSRNOG00000016551	K16341

**Table 7:** Metabolism KEGG pathway

	Pathway	*Q* value	Gene list	KO list
ko00010	Glycolysis/Gluconeogenesis	.038	ENSRNOG00000032095, ENSRNOG00000048095	K00134, K03103
ko00860	Porphyrin and chlorophyll metabolism	.025	ENSRNOG00000011913	K13624
ko00920	Sulfur metabolism	.011	ENSRNOG00000027079	K15759
ko00562	Inositol phosphate metabolism	.002	ENSRNOG00000005284, ENSRNOG00000048095, ENSRNOG00000027079,	K00911, K03103, K15759
ko04659	Th17 cell differentiation	.005	ENSRNOG00000032708, ENSRNOG00000015441, ENSRNOG00000000451	K06752, K05071
ko04658	Th1 and Th2 cell differentiation	.003	ENSRNOG00000032708, ENSRNOG00000015441, ENSRNOG00000000451	K06752, K05071
ko01521	EGFR tyrosine kinase inhibitor resistance	.024	ENSRNOG00000017392, ENSRNOG00000016551	K18497, K16341

**Table 8:** Environmental Information Processing KEGG pathway

Pathway ID	Pathway	*Q* value	Gene list	KO list
ko04070	Phosphatidylinositol signaling system	.025	ENSRNOG00000005284, ENSRNOG00000027079	K00911, K15759
ko04512	ECM–receptor interaction	.024	ENSRNOG00000012471, ENSRNOG00000003357	K04659, K06236
ko04151	PI3K‐Akt signaling pathway	.016	ENSRNOG00000017392, ENSRNOG00000012471, ENSRNOG00000015441, ENSRNOG00000003357, ENSRNOG00000016551	K18497, K04659, K05071, K06236, K16341
ko04514	Cell adhesion molecules (CAMs)	.001	ENSRNOG00000032708, ENSRNOG00000000451, ENSRNOG00000000777, ENSRNOG00000060412, ENSRNOG00000001527	K06752, K06751, K05412

### 3.4 Validation of DEGS

**Figure 4:**
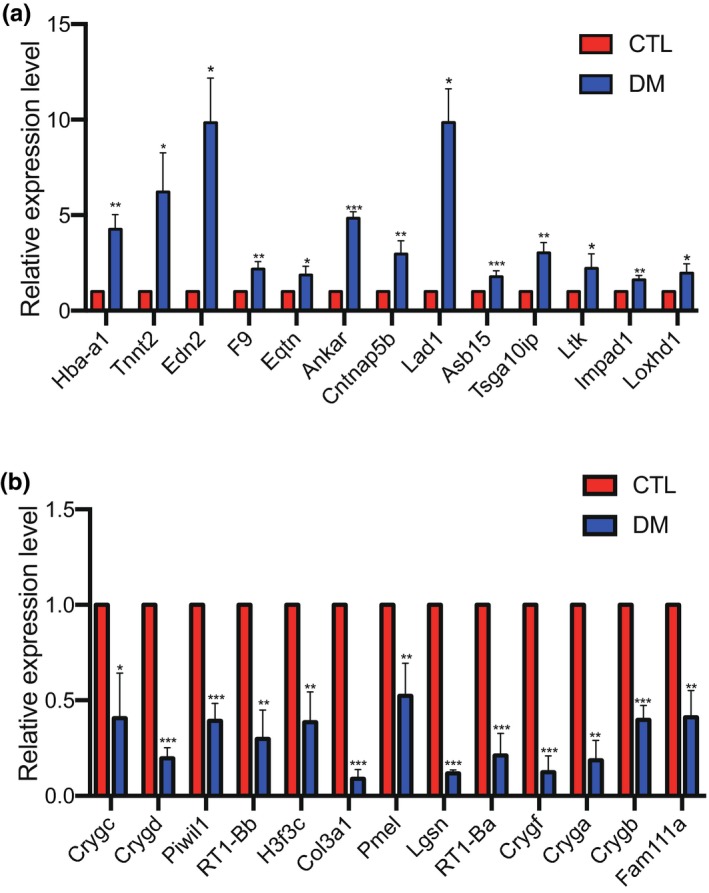
Validation of DEGs. (a) Up‐regulated DEGs. (b) Down‐regulated DEGs. GeneBank ID and version number: Hba‐a1: Gene ID: 287167, Refseq: NC_005109.4 Tnnt2: Gene ID: 24837, Refseq: NC_005112.4 Edn2: Gene ID: 24324, Refseq: NC_005104.4, F9: Gene ID: 24946, Refseq: NC_005120.4, Eqtn: Gene ID: 500502, Refseq: NC_005104.4, Ankar: Gene ID: 501138, Refseq: NC_005108.4, Cntnap5b: Gene ID: 301650, Refseq: NC_005112.4, Lad1: Gene ID: 313325, Refseq: NC_005112.4, Asb15: Gene ID: 500050, Refseq: NC_005103.4, Tsga10ip: Gene ID: 361707, Refseq: NC_005100.4, Ltk: Gene ID: 311337, Refseq: NC_005102.4, Impad1: Gene ID: 312952, Refseq: NC_005116.4, Loxhd1: Gene ID: 291427, Refseq: NC_005117.4, Crygc: Gene ID: 24277, Refseq: NC_005108.4, Crygd: Gene ID: 24278, Refseq: NC_005108.4, Piwil1: Gene ID: 363912, Refseq: NC_005111.4, RT1‐Bb: Gene ID: 309622, Refseq: NC_005119.4, H3f3c: Gene ID: 100360868, Refseq: NC_005106.4, Col3a1: Gene ID: 84032, Refseq: NC_005108.4, Pmel: Gene ID: 362818, Refseq: NC_005106.4, Lgsn: Gene ID: 316304, Refseq: NC_005108.4, RT1‐Ba: Gene ID: 309621, Refseq: NC_005119.4, Crygf, Gene ID: 689947, Refseq: NC_005108.4, Cryga: Gene ID: 684028, Refseq: NC_005108.4, Crygb: Gene ID: 301468, Refseq: NC_005108.4, Fam111a, Gene ID: 499322, Refseq: NC_005100.4

In this study, qRT‐PCR was used to confirm the results of RNA‐seq. Overall, 25 most differentially expressed genes were selected, such as Asb15, Ltk, Eqtn, RT1‐Ba, RT1‐Bb. Changes in the expression levels of these genes were similar to those obtained following RNA‐seq. The complete results are shown in Figure 4.

## 4 DISCUSSION

This study examined retinas from WT and diabetic SD male rats to investigate the changes in a variety of retinal transcripts as a result of diabetes using RNA‐seq. We identified a total of 118 DEGs, of which 72 were up‐regulated and 46 were down‐regulated. We also found 66 GO terms and 41 KEGG pathways which were significantly enriched by GO and KEGG analysis.

Top 10 most down‐regulated and up‐regulated genes are listed in Tables 3 and 4, and were confirmed by qRT–PCR showed in Figure 4. Asb15 gene is the most up‐regulated one we identified and confirmed. Asb15 is a member of Asb gene family; the family has been reported to be involved in cell proliferation and differentiation (Hancock et al., 1991; Kohroki et al., 2001; Liu et al., 2003). The presence of both Ankyrin repeat and suppressors of cytokine signaling (SOCS) box motifs are characters of members of Asb gene family (McDaneld, Hancock, & Moody, 2004). Member of SOCS family plays important roles in the negative regulation of signaling pathways (Kile & Alexander, 2001; Zhang et al., 2001). SOCS3 acts as a regulator of inflammation through inhibiting JAK/STAT pathway (Tamiya, Kashiwagi, & Takahashi, 2011). Down‐regulating SOCS3‐STAT3 can alleviate DR (Chen, Lv, & Gan, 2017; Jiang, Thaksan, & Bheemreddy, 2014; Ye & Steinle, 2015). Ladinin‐1(Lad1), a largely uncharacterized protein to date, was found to be related to the proliferation and migration of breast cancer cells (Roth, Srivastava, & Lindzen, 2018). Cell proliferation and migration are processes of neovascularization. Neovascularization is the sign of PDR, which can lead to serious vision loss of patients. Fibroblast growth factor 2 (Fgf2) is a member of fibroblast growth factors (FGFs) family. FGFs and their receptors have important roles in cell proliferation, migration, differentiation, and survival (Saichaemchan, Ariyawutyakorn, & Varella‐Garcia, 2016). FGF2 was found overexpression in the early stage of DR, and it can destroy the blood–retinal barrier (Yang et al., 2018). Hemoglobin alpha adult chain 1 (Hba‐a1) is one of the hemoglobin genes. Hemoglobin plays an important role in neuronal respiration, oxidative stress, and response to injury (He et al., 2010; Poh, Yeo, Stohler, & Ong, 2012; Richter, Meurers, Zhu, Medvedeva, & Chesselet, 2009). Neuronal respiration is an important life activity of neuronal cells. Neurological injury is one of the performances of DR. Inositol monophosphatase domain containing 1 (Impad1) encodes gPAPP, which is a Golgi‐resident nucleotide phosphatase that hydrolyzes phosphoadenosine phosphate (PAP), the by‐product of sulfotransferase reactions, to AMP. AMP‐activated protein kinase (AMPK) signaling pathway plays vital roles in the diabetes‐induced retinal inflammation (Kubota, Ozawa, & Kurihara, 2011). RT1‐Bb, RT1‐Ba, belongs to RT1 complex, which is the major histocompatibility complex (MHC) of rat (Eberhard & Lutz, 2001). It is believed that the MHC region is vital because it plays an important role in diseases, such as autoimmune and infectious diseases, vascular diseases like DR, hematological and neurological diseases (John, 2005). Collagen type III alpha 1 chain (Col3a1) is a kind of type III collagen, mainly existing in the extracellular matrix. Lacking of type III collagen can destroy the structure of connective tissues (Cortini et al., 2017). According to previous researches, it is associated with the aneurysm. Retinal microaneurysm is the early performance of DR. Col3a1 was also found significantly changed in RNA‐seq of human PDR fibrovascular membranes (Lam et al., 2017). αA‐crystallin (Cryga) and αF‐crystallin (Crygf) are members of crystallins, which were involved in different functions in various tissues (Clayton, Jeanny, Bower, & Errington, 1986; Head, Peter, & Clayton, 1991; Smolich, Tarkington, Saha, & Grainger, 1994). Knockout of αA‐crystallin can inhibit ocular neovascularization (Xu, Bai, & Huang, 2015).

More and more evidence indicated that inflammation (Adamis, 2002; Gologorsky, Thanos, & Vavvas, 2012) and neovascularization (Gardner & Davila, 2017; Nguyen et al., 2018) are important in the pathogenesis of DR. The results of the KEGG pathway significant enrichment analysis revealed two most enrichment items—cell adhesion molecules (CAMs) and PI3K‐Akt signaling pathway. CAMs are proteins located on cell surface; the binding of CAMs to their receptors is important in the mediation of inflammatory and immune reactions (Golias et al., 2007). Previous studies have suggested that CAMs are important in the development of DR (Khalfaoui et al., 2009; Ugurlu et al., 2013). PI3K‐Akt signaling pathway is a main downstream molecular pathway of insulin and is associated with DR neovascularization (Qin, Zhang, & Xu, 2015; Sasore, Reynolds, & Kennedy, 2014).

Compared with the results of transcriptomic analysis which use CD31+ vascular endothelial cells obtained from human PDR fibrovascular membranes (FVM) (Lam et al., 2017), we found the gene Col3a1 were both significantly changed, and according to the GO enrichment analysis of two researches, Col3a1 may belong to blood vessel development term (GO:0001568), suggested that Col3a1 may play a role in DR neovascularization. We also found that the positive regulation of immune system process term (GO: 0002684) was both significantly enriched in two researches. It is a pity that we have not found KEGG analysis of retinas from human DR patients.

According to previous research which used rats' retinas (Yuan et al., 2016), 3 GO terms—biological regulation, response to stimulus, and metabolic processes—were significantly enriched, and KEGG pathway analysis showed that CAMs, complement and coagulation cascades, and antigen processing and presentation, PI3K‐Akt signaling pathway were highly enriched; the above were the same with our research.

Compared with previous similar studies (John, Ram, & Jiang, 2018), less DEGs were identified in this study, which probably due to the individual differences of experimental animals and longer course of diabetes. Previous studies related to DR usually use rats injected with STZ for 2–3 months, we used rats sacrificed 6 months after injecting STZ because DR was definitely occurred at 4–6 months after the development of diabetes (Gong, Lu, & Hu, 2013; Kumar et al., 2013; Si et al., 2013).

## 5 CONCLUSION

Our research identified abnormally expression genes using RNA‐seq, and subsequently, we did GO and KEGG enrichment analysis of those DEGs, which will enhance our understanding of the molecular mechanisms underlying the pathogenesis process of DR. This may allow the development of novel therapeutic targets of DR.

## CONFLICT OF INTEREST

The authors declare that they have no conflict of interest.

## CONSENT FOR PUBLICATION

The authors declare that they consent to publication.
